# Reconsidering Rehabilitation and Lifestyle Approaches to Improve Quality of Life in People with Multiple Sclerosis: A Scoping Review

**DOI:** 10.3390/medicina62010215

**Published:** 2026-01-20

**Authors:** Elena Bianca Basalic, Nadinne Alexandra Roman, Diana Minzatanu, Adina Ionelia Manaila, Ionut Cristian Cozmin Baseanu, Roxana Steliana Miclaus

**Affiliations:** 1 Department of Fundamental, Preventive, and Clinical Disciplines, Faculty of Medicine, Transilvania University of Brasov, 500036 Brasov, Romania; 2 Neurorehabilitation Department, Clinical Hospital of Psychiatry and Neurology, 500036 Brasov, Romania

**Keywords:** multiple sclerosis, rehabilitation, quality of life, lifestyle, self-management, multimodal approaches

## Abstract

*Background*: Multiple sclerosis (MS) involves complex physical, cognitive and behavioral challenges that collectively affect quality of life. Integrating lifestyle components such as sleep optimization, dietary behaviors, stress management, and self-management strategies into rehabilitation may enhance outcomes beyond traditional approaches. This scoping review aimed to map rehabilitation interventions that combine psychomotor, cognitive, lifestyle-focused, or multimodal elements and assess quality of life in adults with MS. *Methods*: This scoping review was conducted in accordance with the PRISMA-ScR guidelines, which guided the identification, screening, and selection of studies. Screening and data extraction were conducted independently by two reviewers. From 135 records, nine primary studies and four secondary evidence sources were included. *Results*: Most studies involved adults with mild-to-moderate disability and predominantly relapsing–remitting multiple sclerosis. Physical or motor rehabilitation interventions were examined in five studies, while two studies evaluated multimodal rehabilitation programs, one study focused on cognitive rehabilitation, and one study investigated lifestyle-oriented or self-management-integrated approaches. Quality of life was assessed in all included studies, with improvements reported across multiple domains. Fatigue-related outcomes were reported in four studies, sleep-related outcomes in three studies, and digitally delivered or hybrid rehabilitation interventions were implemented in three studies, indicating an emerging trend toward technology-supported rehabilitation approaches. *Conclusions*: Contemporary MS rehabilitation is moving toward multidimensional, holistic models that integrate lifestyle components. Standardized outcomes, inclusion of more diverse MS phenotypes, and rigorous evaluation of integrated frameworks are required to strengthen the evidence base and inform clinical practice.

## 1. Introduction

Multiple sclerosis (MS) is a chronic, immune-mediated disorder of the central nervous system (CNS), marked by inflammatory demyelination, axonal injury, and ongoing neurodegeneration. These pathological mechanisms generate widespread lesions whose number, size and anatomical location shape the heterogeneous clinical presentation of MS. People with multiple sclerosis commonly experience motor, cognitive, and mobility impairments, accompanied by a wide range of symptoms that progressively limit daily functioning, participation, and overall quality of life (QoL) [1]. Despite substantial advances in disease-modifying therapies (DMTs) that have improved disease management-through reduced relapse rates, slower lesion accumulation, and delayed disability progression [2], many individuals continue to experience persistent gait disturbances, balance impairments, cognitive dysfunction, and other disabling symptoms that significantly compromise quality of life. These ongoing difficulties may arise from residual CNS damage, chronic inflammatory activity [3] or lifestyle-related behaviors [4]. These persistent difficulties [5] highlight the necessity for additional approaches that address functional impairments insufficiently managed by medication alone [6,7]. Medical rehabilitation offers a comprehensive, integrative framework capable of responding to these unmet needs and represents a key component in limiting the progression of disability.

The literature consistently shows that individuals with MS report lower QOL compared with the general population [8,9]. Health-related quality of life (HRQoL) [10] is understood as a multidimensional construct encompassing physical, emotional, mental, and social well-being [10,11], domains that are frequently affected by this condition. These domains are deeply interconnected and tend to influence one another in complex ways in the context of multiple sclerosis. Beyond disease-related factors, socio-demographic aspects, such as the individual’s educational level, may also shape the way people with MS perceive and experience their quality of life [12]. As the importance of QOL has gained increasing attention, both clinicians and patients have shifted their perspective on what it means to maintain a good life while living with MS [13,14].

Numerous studies have examined the influence of non-pharmacological interventions on QOL and health-related QOL in MS. Such interventions include physical activity [15], psychosocial support, cognitive–behavioral therapy [16], fatigue-management strategies and lifestyle modifications [17,18,19,20]. Diet [21,22], physical activity and sleep can influence MS pathophysiology [23] and consequently modulate fatigue levels [4]. Balanced nutrition supports adequate energy availability and is essential for managing fatigue [24,25]. Regular physical activity increases the production of brain-derived neurotrophic factor (BDNF) [26], which promotes neuronal survival and may help limit neurodegeneration in MS [27].

Exercise also enhances muscle strength and cardiovascular capacity, both of which may reduce physical fatigue frequently experienced by individuals with MS [28]. Adequate sleep plays a crucial role in supporting the body’s restorative functions and can ease physical fatigue, whereas poor or disrupted sleep patterns tend to raise stress-related hormones and inflammatory markers, ultimately worsening fatigue in people with MS [29]. Other studies examining depression [30] in MS have highlighted the harmful consequences of depressive comorbidity [31], such as diminished quality of life and poorer adherence to disease-modifying treatments [32]. Furthermore, early reductions in depressive symptoms have been associated with improvements in fatigue [30], and emerging evidence shows that depression may also heighten the likelihood of disease relapse [33].

Accumulating evidence from clinical and rehabilitation research indicates that the integration of non-pharmacological interventions—including exercise-based programs [34], cognitive and psychological strategies [35], and lifestyle-related approaches [36]—is generally safe and can be effective as a complementary component in the management of multiple sclerosis, particularly with respect to improving quality of life and functional outcomes [37]. However, the existing literature frequently examines these domains separately, without integrating them into a unified perspective on their cumulative impact on quality of life. This observation naturally leads to the central question guiding the present review: *What types of rehabilitation interventions have been used in adults with multiple sclerosis and in what ways do they incorporate lifestyle or self-management components while assessing quality of life?*

Based on this inquiry, the aim of the present review is to provide a synthesis of existing studies in which psychomotor rehabilitation interventions have been combined with lifestyle-focused strategies, such as stress-management or symptom self-management approaches and which have included the assessment of quality of life. This perspective emerged from the intention to leverage, through rehabilitation, the combined potential of factors known for their ability to modulate chronic inflammation. More specifically, this review aims to: (1) classify the types of rehabilitation interventions employed (physical exercise, cognitive therapies, multidimensional programs, lifestyle-focused approaches) to outline recent trends in the field; (2) describe how lifestyle-related elements have been integrated into rehabilitation programs; (3) map the current evidence regarding the reported effects of complex rehabilitation interventions on quality of life; and (4) identify gaps in the existing literature and propose future research directions aimed at developing comprehensive, holistic rehabilitation strategies for people with multiple sclerosis.

## 2. Materials and Methods

### 2.1. Protocol and Reporting Standards

This scoping review was conducted in accordance with the methodological guidance of the Joanna Briggs Institute (JBI) for scoping reviews. Study identification, screening, and selection were performed in line with the Preferred Reporting Items for Systematic Reviews and Meta-Analyses extension for Scoping Reviews (PRISMA-ScR) [38]. The reporting of this review adheres to the PRISMA-ScR checklist, which is provided in Appendix Table A1. A protocol was registered on the Open Science Framework (OSF) in August 2025, following completion of the literature search and prior to study selection, data extraction, and synthesis, to ensure transparency of the review process.

### 2.2. Eligibility Criteria

The study selection process was structured using the PCC framework (Population, Concept, Context), as recommended by JBI [39]. With regard to the population (a), eligible studies included adults (≥18 years) living with multiple sclerosis, without restrictions concerning disease phenotype, disease duration, or level of disability. Concerning the concept (b), studies were required to investigate rehabilitation interventions addressing at least one of the following domains: (1) psychomotor or physical/motor rehabilitation, including balance training, gait rehabilitation, or aerobic exercise; (2) cognitive rehabilitation, encompassing cognitive training or cognitive–behavioral techniques targeting cognitive or emotional outcomes; (3) lifestyle-oriented approaches, such as interventions focused on nutrition, sleep, physical activity, stress management, or symptom self-management; or (4) multimodal interventions combining two or more of these components. Only studies assessing quality of life (QoL) as a primary or secondary outcome were considered eligible. Regarding the context (c), no restrictions were applied, and studies conducted in hospital-based, outpatient, community-based, home-based, or digital and telehealth settings were included. In terms of study design, eligible sources comprised randomized and non-randomized controlled trials, pilot or feasibility studies, quasi-experimental and observational research, as well as secondary evidence, including systematic reviews and meta-analyses. Exclusion criteria included studies involving pediatric populations, studies without a rehabilitation component, studies not reporting quality of life outcomes, validation studies, study protocols without implemented interventions, single-case reports, and conference abstracts without full-text availability.

### 2.3. Data Sources and Search Strategy

A comprehensive search strategy was developed to identify relevant literature examining rehabilitation interventions and lifestyle-related components in adults with multiple sclerosis. Searches were conducted in three electronic databases: PubMed, Web of Science, and ProQuest. The search strategy combined controlled vocabulary (MeSH terms in PubMed) with free-text keywords related to multiple sclerosis, rehabilitation modalities, exercise, cognitive or behavioral interventions, lifestyle factors, self-management strategies, sleep, diet, nutrition, and quality of life. Searches were limited to English-language, peer-reviewed publications to ensure consistency in reporting standards and methodological transparency. The search window covered the period from January 2021 to July 2025, reflecting the increasing focus on lifestyle-oriented and digitally delivered rehabilitation approaches in multiple sclerosis research. The final database search was completed in July 2025. The review protocol was subsequently registered on the Open Science Framework (OSF) in August 2025, prior to study selection, data extraction, and synthesis. Full database-specific search strategies, including Boolean operators for each platform (PubMed, Web of Science, and ProQuest), are provided in Table A2 of the Appendix A.

### 2.4. Study Selection

**Figure 1:**
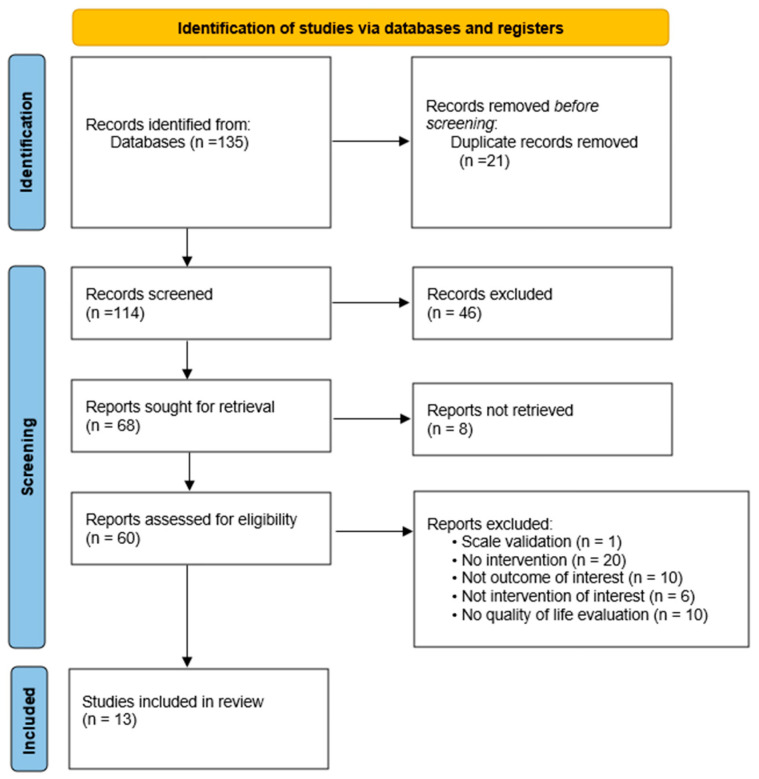
PRISMA diagram flow.

The results obtained from the search were entered into Mendeley Desktop software, version 1.19.8, to remove duplicates. The same program was used to organize the references. The screening process followed a two-step approach. First, titles and abstracts were examined to determine whether records met the predefined eligibility criteria. Subsequently, the full texts of articles deemed potentially relevant were assessed to confirm their suitability for inclusion. Two reviewers conducted the screening independently (E.B.B.; N.A.R.). Disagreements were resolved through discussion, and when consensus could not be reached, a senior reviewer provided the final judgment (R.S.M). A PRISMA flow diagram summarizing the number of records identified, screened, excluded, and ultimately included in the review is provided in Figure 1.

### 2.5. Data Extraction

A structured data charting form was developed to ensure the systematic organization of information extracted from all included evidence sources. Data extraction was conducted separately for primary studies and secondary evidence sources to prevent duplication between original data and synthesized findings. Two reviewers (E.B.B.; N.A.R.) independently read each full-text article and manually extracted the required information using both Mendeley Desktop (version 1.19.8; Elsevier, Amsterdam, The Netherlands) and Microsoft Excel (2024; Microsoft Corporation, Redmond, WA, USA) as complementary charting tools. Extracted entries were compared to ensure accuracy and internal consistency. Disagreements were resolved through discussion; when consensus could not be reached, a senior reviewer (M.S.R) provided the final decision. The charting form was refined iteratively as familiarity with the evidence increased.

### 2.6. Data Items

The following data items were extracted from all included studies, in alignment with the objectives and scope of this scoping review. From primary empirical studies, the extracted data items included: (1) Bibliographic and methodological information: authors, year of publication, country, study design; (2) Population characteristics: sample size, mean or median age, sex distribution, MS subtype, disability level reported by the Expanded Disability Status Scale (EDSS), and relevant baseline descriptors; (3) Intervention characteristics: classification of the rehabilitation approach (psychomotor/physical, cognitive, lifestyle-oriented, or multimodal), detailed description of intervention components, delivery mode (in-person, home-based, digital/telehealth), frequency and duration of the intervention, lifestyle components or self-management strategies integrated into the program; (4) Outcome assessment: instruments used to evaluate quality of life (primary or secondary outcome), and where applicable, tools assessing sleep, fatigue, physical activity, nutrition, stress, or self-management behaviors; (5) Main findings: key results related to quality of life and any lifestyle-related changes reported by the authors.

For systematic reviews and meta-analyses, extracted data items included: (1) Review objectives and scope, including the rehabilitation domains addressed; (2) Characteristics of the evidence synthesized, such as intervention categories and participant profiles reported within the included studies; (3) Quality of life outcomes and lifestyle-related findings highlighted in the review; (4) Authors’ interpretations and aggregated conclusions, without re-extracting data from individual studies. This distinction between primary and secondary evidence sources was applied to avoid duplication of primary findings, as secondary sources may have already synthesized studies independently identified in the present review. (5) Assessment of overlap between primary and secondary evidence sources, with the aim of documenting whether secondary sources included primary studies also identified in the present review, in order to avoid duplication of evidence.

### 2.7. Synthesis of Results

The extracted data were synthesized narratively and organized descriptively in accordance with the objectives of this scoping review. Given the heterogeneity of study designs, populations, and rehabilitation approaches, no meta-analysis or quantitative pooling of results was undertaken, consistent with scoping review methodology. Primary studies were mapped according to the type of rehabilitation intervention employed, psychomotor/physical, cognitive, lifestyle-oriented, or multimodal, to illustrate recent trends and to facilitate comparison across domains. Each category was synthesized in tables outlining participant characteristics, intervention and lifestyle elements, outcomes assessed, and key quality-of-life results. Secondary evidence sources (systematic reviews and meta-analyses) were synthesized in a separate table to prevent overlap with primary studies. These were charted by scope, intervention domains addressed, main conclusions, and overlap with the primary evidence identified in this review. The overall synthesis aimed to provide a structured overview of how rehabilitation and lifestyle-related components have been integrated in recent MS research, without evaluating the methodological quality of included studies, in line with current scoping review guidance. Findings are presented through descriptive tables and narrative summaries that highlight patterns, consistencies, and gaps within the evidence base.

## 3. Results

### 3.1. Overview of the Literature Search

The database search yielded a total of 135 records, which were exported into Microsoft Excel for centralized management. A total of 21 duplicate entries were identified and removed, resulting in 114 unique records subjected to title and abstract screening. During this initial screening, 46 records were excluded for the following reasons: studies conducted in conditions other than multiple sclerosis (*n* = 24); exclusively pharmacological interventions (*n* = 3); pediatric populations (*n* = 1); studies focused on caregivers rather than individuals with MS (*n* = 4); study protocols without implemented interventions (*n* = 13); validation studies (*n* = 1); full text not accessible after repeated attempts (*n* = 8). After this stage, 60 full-text articles were assessed for eligibility. During full-text evaluation, additional records were excluded for reasons aligned with the predefined criteria: scale validation (*n* = 1); absence of an intervention component (*n* = 20); outcomes unrelated to the objectives of this review (*n* = 10); interventions outside the scope of rehabilitation or lifestyle-related components (*n* = 6); lack of quality-of-life assessment (*n* = 10). Following the full-text appraisal, 9 primary studies fulfilled all inclusion criteria. Additionally, 4 secondary evidence sources (systematic reviews or meta-analyses) met eligibility criteria and were synthesized separately to avoid overlap with primary data.

### 3.2. Characteristics of the Included Evidence

The thirteen studies included in this review were published between 2021 and 2025, reflecting the recent emergence of lifestyle-integrated and multidimensional rehabilitation research in multiple sclerosis. The primary evidence base consisted of nine studies employing diverse methodological designs, including randomized controlled trials [40,41,42,43], non-randomized or quasi-experimental interventions [44,45], pilot or feasibility studies [46] and observational pre–post designs [47,48]. The included studies were predominantly conducted in Europe, with contributions from Italy, Denmark, the Netherlands, Germany, and the United Kingdom [40,42,44,46,47,48], alongside research conducted in Australia [43,45], and the United States [47]. Across studies reporting demographic characteristics, participants were mostly female, typically comprising more than two-thirds of each sample. The reported mean ages ranged from the mid-30 s to the late-50 s across interventions. Regarding MS phenotype, most studies included individuals with relapsing–remitting MS [42,43,44,46]. A smaller number of studies enrolled mixed MS populations that included secondary progressive or primary progressive forms [40,47,48]. Baseline disability levels, when reported through the Expanded Disability Status Scale (EDSS), were generally situated within the 1.5–4.5 range across the included studies. Sample sizes ranged from small pilot cohorts to large interventions involving several hundred participants [47]. The secondary evidence base consisted of four studies, comprising one systematic review [49], two systematic reviews with meta-analysis [50,51] and one systematic review incorporating a network meta-analysis [52]. Together they encompassed more than 245 studies and over 18,000 participants. The details and findings are discussed in Table 1.

### 3.3. Intervention Descriptions and Characteristics

The nine primary studies included in this review encompassed four predefined rehabilitation categories: physical/motor rehabilitation, cognitive rehabilitation, lifestyle-oriented interventions and multimodal programs integrating components from two or more domains. The interventions varied substantially in structure, delivery format, duration, and clinical focus, reflecting the heterogeneity of contemporary rehabilitation research in multiple sclerosis.

### 3.4. Physical/Motor Rehabilitation (n = 4 Studies)

Four primary studies investigated physical or motor-focused rehabilitation programs, employing structured exercise-based interventions targeting aerobic capacity, gait efficiency, coordination, and balance [40,44]. Sample sizes ranged from small cohort groups to larger supervised exercise programs, with participants predominantly female and mean ages generally spanning the mid-30 s to the late-50 s. Across studies reporting disease characteristics, most individuals were diagnosed with relapsing–remitting multiple sclerosis and baseline disability levels fell consistently within a mild-to-moderate range (EDSS 1.5–4.5). Intervention modalities reflected substantial diversity. The examined programs included lactate-threshold treadmill aerobic training [44], high-intensity aerobic exercise protocols [40], supervised walking sessions combining continuous and interval-based formats [40], and dance-based motor training using structured Argentine Tango sequences [46]. Targeted motor components varied across interventions: aerobic capacity enhancement was addressed in three studies [40,44] gait-oriented training elements were incorporated in walking-based programs [40] while coordination and balance components were specifically emphasized in the dance-based intervention [46]. Program durations ranged from 12 to 24 weeks, with intervention frequency typically set between one and three supervised sessions per week. Individual sessions lasted approximately 30–60 min, and all interventions were delivered in controlled clinical or community environments; none of the studies implemented home-based or telehealth formats within this category. Quality-of-life outcomes were assessed through validated instruments, including the Multiple Sclerosis Quality of Life-54 (MSQOL-54) [44], the Short Form-36 Health Survey (SF-36) [40,46] and the Multiple Sclerosis Impact Scale-29 (MSIS-29) [40]. Physical capacity, mobility, and fatigue outcomes were measured using standardized tools such as the 6-Min Walk Test (6MWT), maximal oxygen uptake (VO_2_max/VO_2_peak), the Modified Fatigue Impact Scale (MFIS), the Fatigue Severity Scale (FSS), and mobility-specific PROMs including the Multiple Sclerosis Walking Scale-12 (MSWS-12). None of the included physical rehabilitation studies explicitly reported adherence or dropout data.

### 3.5. Cognitive Rehabilitation (n = 1 Study)

One study investigated a cognitive rehabilitation approach targeting cognitive and emotional functioning in adults with multiple sclerosis [42]. The sample consisted of adults with relapsing–remitting MS, predominantly female, with mild-to-moderate disability levels; baseline characteristics, including age and EDSS scores, were reported within ranges comparable to other included interventions. The study evaluated two structured cognitive programs: an eight-week Mindfulness-Based Cognitive Therapy (MBCT) intervention and a nine-week Cognitive Rehabilitation Therapy (CRT) protocol, each compared with a psychoeducation control condition. The MBCT program incorporated mindfulness meditation practices, attentional regulation exercises, and emotional awareness tasks designed to modulate stress-related cognitive responses. The CRT intervention applied compensatory and restorative cognitive strategies aimed at improving domains such as memory, attention, and executive functioning. Interventions were delivered in supervised group-based formats, with weekly sessions supported by home practice components. Quality of life outcomes were assessed using the Multiple Sclerosis Quality of Life-54 (MSQOL-54), while additional cognitive and emotional domains were measured with instruments including the Hospital Anxiety and Depression Scale (HADS), the Cognitive Failures Questionnaire (CFQ), and the Five Facet Mindfulness Questionnaire (FFMQ). Reported outcomes indicated improvements in depression, mindfulness, and mental quality of life indices, with the MBCT arm additionally showing reductions in fatigue-related cognitive complaints.

### 3.6. Lifestyle-Oriented Interventions (n = 1 Study)

One study examined a lifestyle-oriented rehabilitation program delivered through a structured telehealth format [45]. The intervention targeted adults with multiple sclerosis presenting with mild-to-moderate disability levels, predominantly women, with mean ages situated within the typical adult MS range. Baseline characteristics, including MS phenotype and EDSS scores, were reported in alignment with the broader sample profiles of the included primary studies. The program integrated multiple lifestyle components, combining fatigue management education, sleep hygiene strategies, energy conservation techniques, diaphragmatic breathing, relaxation and mindfulness exercises, alongside daily step monitoring. The intervention was implemented over a five-week period, and participants underwent additional follow-up assessments at three and six months to capture sustained behavioral changes. Lifestyle-related and health status outcomes were assessed using standardized tools, including the Fatigue Severity Scale (FSS), the Pittsburgh Sleep Quality Index (PSQI), and health-related quality-of-life measures derived from the EuroQol 5-Dimension 3-Level Questionnaire (EQ-5D-3L) and the EuroQol Visual Analogue Scale (EQ-VAS). Objective physical activity data were recorded through daily step counts collected across the intervention period. Reported outcomes indicated reductions in fatigue levels, improvements in sleep quality, and increases in daily physical activity, with several effects maintained at follow-up evaluations.

### 3.7. Multimodal Rehabilitation (n = 3 Studies)

Three studies evaluated multimodal rehabilitation programs that combined psychomotor or physical components with lifestyle-oriented and/or cognitive-emotional strategies [43,47,48]. Saxby et al., 2024 [47] conducted a prospective pilot study including 44 adults with clinically isolated syndrome or relapsing–remitting MS who were within 12 months of diagnosis and voluntarily disease-modifying therapy-naive. The sample was predominantly female, with a mean age in the late 30 s to early 40 s. Participants received a 12-month remotely delivered multimodal program consisting of a modified Paleolithic diet, a structured walking regimen of 150 min per week, 4–7–8 breathing exercises and monthly wellness coaching sessions, compared with a standard-of-care group receiving disease-modifying treatment. Quality of life was assessed using the Multiple Sclerosis Quality of Life-54 (MSQOL-54), while fatigue and perceived cognitive difficulties were evaluated with the Modified Fatigue Impact Scale (MFIS), Fatigue Severity Scale (FSS) and the Perceived Deficits Questionnaire (PDQ). Mood and anxiety were measured with the Hospital Anxiety and Depression Scale (HADS), and physical activity with the International Physical Activity Questionnaire Long Form (IPAQ-Long). Over 12 months, the multimodal lifestyle group showed mean changes in fatigue, quality of life, mood and cognition that were not inferior to those observed in the standard-of-care group; within-group analyses indicated improvements in depressive symptoms, fatigue scores and PDQ total scores in the intervention arm.

Meyer et al., 2024 [43] reported a large parallel-group randomized controlled trial (DiQoLiMS) including 421 adults with MS (relapsing–remitting, secondary progressive and primary progressive phenotypes) diagnosed for at least one year (mean age 47.5 years; 78–79% women). Participants were randomized to standard care plus levidex (a comprehensive CBT-based digital lifestyle management program) or to an active control receiving standard care plus web-adapted lifestyle information from the national MS society. The levidex intervention delivered modular content on physical activity, diet, sleep, stress management, emotional regulation and health-behavior change techniques over 6 months. The primary outcome was MS-related quality of life measured with the Hamburg Quality of Life Questionnaire in MS (HAQUAMS); secondary outcomes included the World Health Organization-Five Well-Being Index (WHO-5), the Multiple Sclerosis Walking Scale-12 (MSWS-12), the Frenchay Activity Index (FAI), the short Dietary Quality Screener (sDQS), the Food Quality Questionnaire (FQQ) and days of sick leave. At 6 months, the intervention group showed statistically significant improvements in overall MS-related quality of life (HAQUAMS total score), with additional benefits on cognitive and mood subscales, together with fewer self-reported sick days and higher activity levels on the Frenchay Activity Index, while the fatigue subscale of HAQUAMS demonstrated a trend toward improvement.

D’Arma et al., 2022 [48] presented a pre–post multimodal residential program including 15 people with MS (8 men, 7 women; mean age 49.1 years), with a mix of relapsing–remitting (*n* = 10) and secondary progressive (*n* = 5) forms and a mean Expanded Disability Status Scale (EDSS) score of 5.4 (range 2–8). The Brief High-Impact Preparatory Experience (B-HIPE) combined neuromotor rehabilitation with a Mediterranean-style dietary regimen, mindfulness practice, adapted sailing sessions and socio-cultural group activities delivered in a seaside camp setting over a short intensive period. Clinical and patient-reported outcomes included the 36-Item Short Form Survey (SF-36) for quality of life, the Medical Outcomes Study Sleep Scale (MOSS), the Epworth Sleepiness Scale (ESS), the International Restless Legs Syndrome Scale (IRLSS) and the Hospital Anxiety and Depression Scale (HADS). In addition, neuroendocrine markers such as β-endorphins, noradrenaline, dopamine, cortisol and serotonin were assessed by ELISA. Post-intervention analyses indicated significant improvements in several SF-36 domains (role limitations due to physical and emotional problems, energy/fatigue, emotional wellbeing, social functioning and pain), better subjective sleep quality (MOSS, ESS) and reduced anxiety symptoms, accompanied by increased β-endorphin and noradrenaline levels. Taken together, these three multimodal programs combined structured rehabilitation with diet, sleep or stress-management elements and systematically assessed quality of life alongside fatigue, mood, sleep or activity-related outcomes. More detailed information on sample characteristics, intervention components, outcome measures and reported results for each study is presented in Table 2.

### 3.8. Summary of Included Secondary Evidence

Four secondary evidence sources were included in this review, comprising systematic reviews, one systematic review with meta-analysis, and one systematic review incorporating a network meta-analysis. Each provides a broader synthesis of the literature on lifestyle behaviors, rehabilitation modalities, and symptom management interventions in multiple sclerosis. The following subsections present a structured, narrative analysis of each evidence source, detailing methodological characteristics, scope, intervention categories, sample sizes, outcomes assessed and key conclusions.

Wills and Probst, 2022 [50] conducted a comprehensive systematic review and meta-analysis evaluating the effectiveness of lifestyle-based self-management strategies on quality of life and disability outcomes in multiple sclerosis. The review synthesized findings from 57 studies, encompassing approximately 5830 participants, and focused on interventions such as multicomponent lifestyle programs, physical activity, diet-related strategies, stress-management techniques, and self-guided behavioral programs. Interventions were heterogeneous, ranging from structured group programs to remote or digital self-management strategies. Quality-of-life outcomes were frequently assessed using tools such as the MSQOL-54, SF-36, and MSIS-29, while disability measures included EDSS, Patient-Determined Disease Steps (PDDS), relapse rate, and MRI-based indicators. The authors reported that multicomponent lifestyle self-management programs were consistently associated with improvements in quality of life, whereas the effects of physical activity alone were mixed, largely due to methodological variability across trials. Dietary interventions showed inconsistent or modest effects when implemented in isolation. The review did not overlap with any of the primary studies included in this scoping review.

Belveal et al., 2023 [49] performed a systematic review examining nontraditional and home-based rehabilitation interventions designed to improve activities of daily living (ADLs) and functional capacity in adults with multiple sclerosis. The review included 15 studies with a combined sample of 782 participants, encompassing modalities such as home-based strengthening programs, vestibular rehabilitation, yoga, music-supported movement training, and digital cognitive–behavioral therapy platforms. The primary outcomes in this review targeted ADL performance, balance, mobility, dexterity, and fatigue, with secondary attention to psychological well-being. The authors identified strong evidence supporting the effectiveness of nontraditional and home-based rehabilitation, particularly programs incorporating either embodied movement approaches (e.g., vestibular or yoga interventions) or digital self-management formats (e.g., web-based CBT modules). Improvements were frequently captured through functional tests, self-reported functional scales, and task-specific performance measures. Similarly to the other secondary sources, no overlap with the primary studies of this review was identified.

Dias et al., 2020 [51] conducted a systematic review and meta-analysis assessing whether exercise-based telerehabilitation enhances pain, physical function, and quality of life in individuals with physical disabilities, including multiple sclerosis. The review encompassed 60 randomized controlled trials, of which 48 were pooled in the meta-analysis, accounting for approximately 4920 participants across diagnostic groups. Although the review was not MS-specific, a substantial subset of included trials involved MS populations, allowing indirect insight into remote rehabilitation trends. Interventions consisted of synchronous or asynchronous telerehabilitation platforms delivering aerobic exercise, resistance training, balance training, or mixed exercise programs. Quality-of-life outcomes were primarily measured using the SF-36 and diverse patient-reported outcome measures. The meta-analysis concluded that exercise-based telerehabilitation is not inferior to traditional face-to-face rehabilitation for improving pain, physical function, and health-related quality of life. Evidence comparing telerehabilitation to no-treatment or usual-care control groups was more limited but suggested potential benefits. No overlap with primary studies was detected.

Harrison et al., 2021 [52] conducted a large-scale systematic review combined with a network meta-analysis to determine which exercise, behavioral, or combined interventions most effectively reduce fatigue in multiple sclerosis. The review included 113 studies with a total sample size of 6909 participants, covering a wide spectrum of rehabilitation modalities such as aerobic exercise, resistance training, balance training, mindfulness, cognitive–behavioral therapy, and multimodal interventions. Fatigue outcomes were typically measured using the Modified Fatigue Impact Scale (MFIS), the Fatigue Severity Scale (FSS), and additional validated self-report instruments. The network meta-analysis identified balance-oriented exercise programs and cognitive–behavioral therapy as ranking highest in terms of efficacy, followed by moderate evidence supporting aerobic and resistive exercise. Combined interventions appeared promising but were underrepresented in the literature. Follow-up data suggested that some interventions sustained improvements beyond the immediate post-intervention period. As with the other secondary sources, no overlap with primary studies included in the present review was observed. More detailed information is available in Table 3.

## 4. Discussion

This scoping review aimed to map contemporary rehabilitation approaches that integrate psychomotor, cognitive, lifestyle-related, or multimodal components and that evaluate quality of life in adults with multiple sclerosis. The synthesis of evidence revealed a growing shift toward multidimensional rehabilitation frameworks, in which traditional physical exercise programs are increasingly combined with dietary guidance, sleep optimization strategies, stress-management techniques or self-management training. Across the nine primary studies, most interventions targeted adults with mild-to-moderate disability and relapsing–remitting MS, and all assessed quality of life using validated instruments, although heterogeneity in outcome measures limited direct comparability across studies. The findings showed that lifestyle-oriented and multimodal interventions, particularly those delivered digitally or through hybrid formats, yielded improvements across several domains including fatigue, mood, sleep quality and perceived functioning, suggesting that rehabilitation strategies incorporating lifestyle components may address dimensions insufficiently targeted by exercise-based protocols alone.

Previous reviews published prior to 2021 provide an important framework for interpreting the present findings. Earlier evidence consistently highlighted the beneficial role of rehabilitation and self-management-oriented interventions in improving quality of life and psychosocial outcomes in people with multiple sclerosis. For example, Kidd et al., 2017 [53] reported that self-management interventions demonstrated favorable effects on health-related quality of life in most included randomized controlled trials, although the evidence was limited by small sample sizes, heterogeneity of interventions, and incomplete reporting of intervention components, which constrained comparability and generalizability of findings. Similarly, Gil-González et al., 2020 [54] synthesized a broad body of literature emphasizing that quality of life in multiple sclerosis is strongly influenced by a complex interplay of clinical factors—such as disability, fatigue, and disease progression-and psychosocial variables, including depression, anxiety, coping strategies, self-efficacy, resilience, and social support. Their findings underscored the relevance of psychological and self-management-based interventions, including mindfulness, cognitive behavioral approaches, and structured self-help programs, in improving multiple dimensions of quality of life. In addition, earlier narrative reviews focusing on the role of rehabilitation emphasized the importance of engagement, participation, and balance in daily life as key targets of multidisciplinary rehabilitation programs. For instance, Peeters et al., 2020 [55] highlighted that rehabilitation in multiple sclerosis extends beyond symptom reduction, aiming to optimize meaningful participation and long-term well-being, while also identifying persistent gaps related to outcome standardization, integration of lifestyle components, and long-term evaluation of intervention effectiveness. Taken together, earlier reviews established a solid foundation demonstrating that non-pharmacological and rehabilitation-based interventions positively influence quality of life and psychosocial functioning in multiple sclerosis. However, they also consistently pointed to substantial methodological heterogeneity and a fragmented approach to lifestyle integration, providing the context against which more recent, multimodal, and lifestyle-oriented rehabilitation frameworks have emerged.

Several recent reviews have further expanded the evidence base regarding non-pharmacological and rehabilitation-based interventions in multiple sclerosis, providing an important context for interpreting the present findings. A recent systematic review and meta-analysis by Du et al., 2024 [56], demonstrated significant beneficial effects of exercise interventions on balance, walking ability, fatigue, and quality of life, with aerobic and resistance training emerging as particularly effective modalities. These findings are consistent with the predominance of exercise-based interventions identified in our review, especially with respect to improvements in functional outcomes and quality-of-life.

Similarly, Gitman et al., 2023 [57] synthesized randomized controlled trials evaluating a broad range of non-pharmacological interventions and reported modest but consistent improvements in both physical and mental components of quality of life, particularly for interventions incorporating physical activity and balance training. While their quantitative synthesis focused primarily on effect sizes across intervention categories, our review complements these findings by qualitatively examining how physical, cognitive, and lifestyle-oriented components are combined within multimodal rehabilitation frameworks.

Narrative and mini-reviews have also highlighted the expanding scope of rehabilitation in multiple sclerosis. Faraclas, 2023 [37] emphasized the growing importance of non-pharmacological strategies such as exercise, cognitive behavioral therapy, and cognitive rehabilitation in promoting holistic quality of life outcomes across physical, emotional, and social domains. In parallel, Duan et al., 2023 [58] underscored the increasing role of emerging rehabilitation technologies, including transcranial magnetic stimulation, virtual reality, robot-assisted gait training, and telerehabilitation, as promising adjuncts to conventional rehabilitation approaches.

Collectively, these recent reviews converge in demonstrating the positive impact of rehabilitation and non-pharmacological interventions on quality of life in people with multiple sclerosis. In contrast to reviews that focus on single intervention domains or quantitative effect estimation, the present scoping review extends the literature by mapping the integration of physical, cognitive, and lifestyle-related components within multimodal rehabilitation approaches and by identifying persistent heterogeneity and gaps that limit comparability and long-term implementation.

We acknowledge that several of the overarching conclusions of the present review particularly regarding the positive effects of exercise-based and non-pharmacological interventions on quality of life in people with multiple sclerosis- have been consistently reported in previous reviews [34,54,57]. This convergence of findings reinforces the robustness of the existing evidence base and underscores the established role of rehabilitation in supporting quality of life outcomes.

However, the present scoping review contributes novel insights by shifting the analytical focus from isolated intervention modalities toward the structure and integration of multimodal rehabilitation approaches. Specifically, our review highlights the increasing incorporation of lifestyle-related components—such as physical activity, cognitive and psychological strategies, and self-management elements—within rehabilitation frameworks, extending beyond exercise alone. In addition, by synthesizing both primary and secondary evidence, the present review identifies substantial heterogeneity in intervention composition, outcome selection, and follow-up duration, emphasizing the need for greater standardization and more robust long-term evaluation of integrated rehabilitation models in multiple sclerosis.

In addition to the interventions identified in the present review, several rehabilitation domains remain insufficiently explored in relation to quality-of-life outcomes in people with multiple sclerosis. Breathing and respiratory-focused exercises, which have demonstrated benefits in other neurological and chronic conditions, may represent a relevant adjunct to comprehensive rehabilitation programs, particularly given their potential impact on fatigue, anxiety, autonomic regulation, and perceived well-being. However, evidence directly linking structured breathing interventions to quality-of-life outcomes in multiple sclerosis remains limited and warrants further investigation. Moreover, the recent literature has highlighted the growing interest in emerging rehabilitation technologies, including virtual reality-based interventions, robot-assisted gait training, non-invasive brain stimulation techniques such as transcranial magnetic stimulation, and digitally delivered or tele-rehabilitation approaches [58,59]. Although these modalities were not sufficiently represented among the primary studies included in the present review, they hold promise for enhancing engagement, personalization, and accessibility of rehabilitation, and may play an important role in future multimodal and lifestyle-integrated rehabilitation frameworks aimed at improving quality of life in people with multiple sclerosis.

When these results are positioned within the broader literature, several areas of convergence become evident. Previous research has consistently shown that lifestyle-related behaviors, including sleep quality, stress regulation, physical activity, and dietary patterns, influence symptom burden, functional capacity, and inflammatory processes in multiple sclerosis [28]. Evidence from recent scoping reviews and observational cohorts similarly indicates that structured lifestyle programs can improve fatigue, emotional well-being, and daily functioning [60,61]. The benefits observed in multimodal interventions are consistent with earlier studies demonstrating that combined approaches integrating exercise with cognitive–behavioral and lifestyle-support components yield greater improvements than single-domain interventions. In particular, the positive effects associated with mindfulness- and stress-management-based strategies in the included studies align with previous findings highlighting the relevance of emotional and behavioral domains in rehabilitation outcomes [9].

Although physical exercise represented a core component of many interventions included in this review, objective assessment of physical capacity was rarely performed using cardiopulmonary exercise testing (CPET), which is widely regarded as the gold standard for evaluating aerobic capacity and exercise tolerance in both clinical and research settings. Recent evidence, including the NOODLE study, has highlighted the value of CPET in accurately characterizing physiological responses to exercise and guiding individualized exercise prescription in people with multiple sclerosis [62]. The limited use of CPET across rehabilitation studies may contribute to heterogeneity in exercise dosing and outcome interpretation and represents an important methodological gap. Future rehabilitation research would benefit from incorporating CPET-based assessment to improve standardization, optimize intervention design, and strengthen the interpretability of exercise-related outcomes.

Across the included studies, several conceptual patterns emerged. Interventions increasingly targeted symptom clusters rather than isolated impairments, reflecting a shift toward rehabilitation models that acknowledge the interdependence of fatigue, mood, sleep, and cognitive functioning. Lifestyle-related components were progressively integrated into rehabilitation programs, consistent with evidence suggesting that behavioral and environmental factors modulate neuroinflammatory processes and functional outcomes. The growing adoption of digital and telehealth-based delivery formats reflects a broader trend toward technology-enabled rehabilitation and may offer advantages for individuals facing mobility limitations or geographic barriers.

Despite these advances, important gaps in the current evidence base remain. Cognitive rehabilitation was underrepresented relative to its clinical relevance, and dietary interventions were seldom evaluated despite increasing interest in the potential role of nutrition in modulating inflammation and fatigue. Many studies lacked detailed reporting on intervention adherence, fidelity, and long-term follow-up, limiting conclusions regarding the sustainability of observed effects. Furthermore, individuals with progressive multiple sclerosis or higher disability levels were minimally represented, a limitation also documented in broader rehabilitation research [58,59]. Objective assessments of lifestyle-related domains, such as sleep metrics, physiological stress markers, or standardized measures of diet quality, were used infrequently, and the predominance of studies conducted in high-income settings restricts the generalizability of findings to more diverse sociocultural contexts.

These gaps highlight several priorities for future research. Large, multisite trials are needed to evaluate integrated rehabilitation models and to incorporate objective behavioral and biological measures in order to better elucidate underlying mechanisms of change. In addition, greater standardization of quality-of-life and lifestyle-related outcomes would improve comparability across studies and facilitate the identification of consistent patterns. Expanding recruitment to include individuals with progressive disease phenotypes and higher levels of disability is essential to improve representativeness. Further research should also focus on optimizing digital delivery models and developing dietary interventions grounded in mechanistic insights. Clinically, the available evidence suggests that incorporating lifestyle-related components into rehabilitation programs may enhance outcomes beyond those achieved through traditional physical training alone. Multidomain rehabilitation frameworks that integrate exercise with sleep optimization, stress-management strategies, nutritional guidance, and cognitive or behavioral interventions may be particularly beneficial for individuals experiencing complex symptom profiles.

Taken together, the findings of this scoping review indicate that contemporary rehabilitation for multiple sclerosis is evolving toward holistic, interconnected approaches that integrate physical, cognitive, emotional, and lifestyle dimensions to improve quality of life. Although the current evidence base remains heterogeneous and methodologically variable, the observed patterns underscore the potential of integrated, lifestyle-informed rehabilitation strategies. By identifying current trends, consistencies with the broader literature, and persistent gaps, this review provides a foundation for advancing comprehensive rehabilitation programs capable of addressing the multifaceted needs of people living with multiple sclerosis.

**Figure 2:**
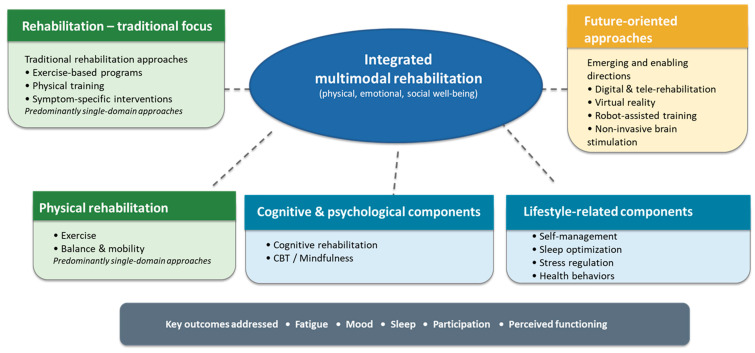
Evolution toward integrated, lifestyle-informed rehabilitation approaches for improving quality of life in multiple sclerosis. The figure summarizes the shift from traditional, single-domain rehabilitation toward integrated multimodal approaches that combine physical, cognitive, psychological, and lifestyle-related components to address key quality-of-life outcomes. Emerging and future-oriented rehabilitation strategies are also highlighted.

*Clinical implications* from this scoping review emphasize the importance of integrated rehabilitation approaches in multiple sclerosis. While physical exercise remains a central component of rehabilitation, combining psychomotor training with lifestyle-related strategies—such as fatigue management, sleep optimization, stress regulation, and cognitive or behavioral support—appears to enhance quality-of-life outcomes. Multimodal and digitally supported interventions may be particularly effective in addressing complex symptom profiles and improving accessibility to rehabilitation services. These findings support the adoption of holistic, patient-centered rehabilitation programs in which quality of life represents a key clinical outcome guiding intervention design and evaluation. To facilitate interpretation for clinicians and readers, Figure 2 summarizes the evolution toward integrated, lifestyle-informed rehabilitation approaches highlighted in this review.

## 5. Limitations

Several limitations of this scoping review should be acknowledged. First, restricting the search to English-language publications may have resulted in the exclusion of relevant evidence produced in non-English-speaking settings, potentially limiting the cultural and geographical diversity of the included studies. Similarly, although the decision to focus on the 2021–2025 period was justified by the recent expansion of lifestyle-integrated and multidimensional rehabilitation models, this timeframe may have excluded earlier foundational work that could have informed long-term trends in the field. Second, the substantial heterogeneity across study designs, intervention protocols, delivery formats and outcome measures constrained the ability to identify consistent patterns of effectiveness. In addition, as this study was designed as a scoping review, no formal quality assessment of the included studies was performed, which represents a limitation when interpreting the methodological robustness of the available evidence. Variability in reporting quality, particularly regarding intervention fidelity, adherence and retention rates, follow-up assessments and detailed characterization of clinical profiles such as MS phenotype, disease duration, or disability level, also limited the depth of comparative synthesis and reduced interpretability. These reporting inconsistencies mirror concerns frequently noted in the broader rehabilitation literature [63,64]. Third, most included studies were conducted in high-income countries with well-developed rehabilitation infrastructures, which may restrict the transferability of findings to regions with different sociocultural, economic, or healthcare contexts. This imbalance highlights a persistent gap in global representation and may affect the generalizability of lifestyle-integrated rehabilitation strategies. Finally, the absence of standardized operational definitions for “multimodal,” “lifestyle-based,” or “integrative” rehabilitation across studies posed challenges for classification and synthesis. Inconsistencies in how authors described or combined intervention components complicated comparisons and underscore the need for more systematic reporting frameworks in future research.

## 6. Conclusions

This scoping review mapped contemporary rehabilitation approaches that integrate psychomotor, cognitive, lifestyle-related, or multimodal components to improve quality of life in adults with multiple sclerosis. The findings indicate a clear shift toward multidomain rehabilitation frameworks in which traditional physical training is complemented by behavioral, emotional, and lifestyle-oriented strategies. Interventions incorporating elements such as sleep optimization, stress management, dietary guidance, or self-management were associated with improvements in fatigue, mood, sleep quality, and perceived functioning. However, substantial heterogeneity in intervention design, study methodology, and outcome measures limits comparability across studies and highlights the need for greater standardization and long-term evaluation. By synthesizing both primary studies and secondary evidence while analyzing them separately, this review provides a comprehensive and methodologically coherent overview of recent developments in the field. Overall, the findings support the growing relevance of holistic, lifestyle-informed rehabilitation strategies and underscore the need for future research to adopt standardized outcomes, include diverse MS phenotypes, and rigorously evaluate digital and hybrid delivery models.

## Figures and Tables

**Table 1:** **Study and population characteristics.**

Author, Year	Study Design	Sample Size	Age (Mean ± SD)	Sex	MS Type	EDSS	Disease Duration
Amato et al., 2021 [44]	Pre–post interventional aerobic study	20	45.8 ± 10.2	13 F/7 M	RRMS	2.5 ± 1.2	12.1 ± 7.9 yrs
Langeskov-Christensen et al., 2022 [40]	RCT (PAE vs. waitlist)	86	EX: 44.0 ± 9.5; CTRL: 45.6 ± 9.3	~60% F	RRMS 87.2%; PP 12.8%	2.7 ± 1.4/2.8 ± 1.6	10.9 ± 7.9/8.6 ± 6.0 yrs
Trinchillo et al., 2024 [46]	Observational pre–post pilot	7	41.14 ± 14.27	6 F/1 M	Early RRMS	Median 2.1 (range 1–3)	14.14 ± 7.6 yrs
Hvid et al., 2025 [41]	RCT	58 (35 EX, 23 CTRL)	EX: 47 ± 9; CTRL: 48 ± 10	74% F	RRMS	2.8 ± 1.5	11.5 ± 7.4 yrs
Nauta et al., 2024 [42]	RCT (MBCT, CRT, ETAU)	99	48.8 ± 9.6	74% F	RR 64%; SP 18%; PP 13%; unclassified 5%	Median 4.0 (range 2–7.5)	Median 8 yrs (3–19.2)
Yalçın et al., 2025 [45]	Quasi-experimental repeated-measures	30	34.3 ± 8.1	80% F	RRMS	2.4 ± 1.8	8.8 ± 6.3 yrs
Saxby et al., 2024 [47]	Prospective pilot	44	38–41	Mostly F	CIS/RRMS	NR	≤12 months since diagnosis
Meyer et al., 2024 [43]	RCT	421	47.4 ± 10.3	78.6% F	RR, SP, PP	NR	≥1 year since diagnosis
d’Arma et al., 2022 [48]	Pre–post multimodal program	15	49.13 ± 8.52	8 M/7 F	RR = 10; SP = 5	5.4 ± 1.66	19.38 ± 5.05 yrs

RRMS = relapsing–remitting multiple sclerosis; CIS = clinically isolated syndrome; EDSS = Expanded Disability Status Scale; yrs = years; EX = exercise group; CTRL = control group; F = female; M = male; MBCT = mindfulness-based cognitive therapy; CRT = cognitive rehabilitation therapy; ETAU = enhanced treatment as usual; mo = months; RCT = Randomized controlled trial; NR = not reported.

**Table 2:** **Intervention characteristics and outcomes.**

Author, Year	Intervention Type	Intervention Description	Duration	Lifestyle Components Integrated	Assessment Instruments	Main Results
Amato et al., 2021 [44]	Physical/Motor Rehabilitation	Lactate-threshold aerobic treadmill training; individualized HR zones; progressive load	12 wks	PA	MSQOL-54, FSS, HADS, 6MWT, LT test	↑ Physical & mental QoL; ↓ fatigue; ↑ walking distance; ↑ aerobic threshold
Langeskov-Christensen et al., 2022 [40]	Physical/Motor Rehabilitation	High-intensity aerobic exercise (supervised): 2×/week; 30–60 min; 65–95% HRmax; continuous + interval cycling/rowing	24 wks	None	MFIS, FSS, MSWS-12, 6MWT, VO_2_max, SF-36	↓ Fatigue impact; ↑ aerobic capacity; small ↑ walking; no change in SF-36
Trinchillo et al., 2024 [46]	Physical/Motor Rehabilitation (Dance-based)	Tango therapy: 1 h/week × 20 weeks; warm-up + technique + partner dance; supervised + home practice videos	20 wks	None	Zung, BDI-II, MSNQ, MSISQ, BBS, Tinetti, FSS, SF-36	↑ Mood, ↑ sexual satisfaction, ↑ perceived cognition, ↑ QoL; stable balance
Hvid et al., 2025 [40]	Physical/Motor Rehabilitation	Supervised group walking exercise; continuous + interval walking; intensity progression; outdoor supervised	24 wks	None	MFIS, FSS, 6MWT, MSWS-12, VO_2_peak, MSIS-29, HADS	↓ Fatigue (MFIS), ↑ VO_2_peak, ↑ walking capacity; no changes in anxiety/depression
Nauta et al., 2024 [42]	Cognitive Rehabilitation (MBCT & CRT)	MBCT: 8 × 2.5 h sessions + 5 h retreat + daily practice; CRT: 9-week compensatory cognitive training; ETAU = psychoeducation	9 wks + FU	None	HADS, CIS-20, RRS, MSQOL-54, FFMQ, SCS, USER-P	↓ Depression; MBCT ↓ fatigue & brooding; ↑ mindfulness; ↑ mental QoL
Yalçın et al., 2025 [45]	Lifestyle (Sleep + PA + Fatigue mgmt)	Telehealth fatigue & sleep education; energy conservation; breathing exercises; relaxation; mindfulness; step tracking	5 wks + 3 & 6 mo FU	Sleep, PA, stress mgmt, fatigue mgmt	FSS, PSQI, EQ-5D-3L, EQ-VAS, step count	↓ Fatigue; ↑ sleep quality; ↑ PA; sustained changes at follow-up
Saxby et al., 2024 [47]	Multimodal (Lifestyle + PA + Stress mgmt)	Modified Paleolithic diet; walking 150 min/week; 4–7–8 breathing; wellness coaching (remote + monthly sessions)	12 mo	Diet, PA, stress mgmt, self-management	MSQOL-54, MFIS, FSS, IPAQ-Long, PDQ, HADS	↑ Mental QoL; ↓ fatigue; ↑ cognition; ↓ anxiety; SOC ↑ for depression & physical QoL
Meyer et al., 2024 [43]	Multimodal (CBT + Lifestyle)	Digital CBT-based lifestyle program: modules for PA, diet, sleep, stress mgmt, emotional regulation, behavior change	6 mo	Diet, PA, sleep, stress, CBT	HAQUAMS, WHO-5, MSWS-12, FAI, sDQS, FQQ	↑ QoL; ↑ mood; ↑ cognition; ↓ sick days; trend ↓ fatigue
d’Arma et al., 2022 [48]	Multimodal (Physical + Lifestyle + Cognitive-emotional)	Neuromotor rehab; Mediterranean diet principles; mindfulness; adapted sailing; group socio-cultural activities	Intensive short program	Diet, PA, mindfulness, wellbeing	SF-36, HADS, MOSS, ESS, IRLSS; β-endorphin, noradrenaline, dopamine, serotonin, cortisol	↑ QoL; ↑ sleep; ↓ anxiety; ↑ β-endorphin & noradrenaline; neuroendocrine modulation

PA = physical activity; QoL = quality of life; MBCT = mindfulness-based cognitive therapy; CRT = cognitive rehabilitation therapy; CBT = cognitive behavioral therapy; MFIS = Modified Fatigue Impact Scale; FSS = Fatigue Severity Scale; MSQOL-54 = Multiple Sclerosis Quality of Life-54; MSWS-12 = Multiple Sclerosis Walking Scale-12; 6MWT = Six-Minute Walk Test; HADS = Hospital Anxiety and Depression Scale; PSQI = Pittsburgh Sleep Quality Index; IPAQ-Long = International Physical Activity Questionnaire-Long Form; PDQ = Perceived Deficits Questionnaire; HAQUAMS = Hamburg Quality of Life Questionnaire in MS; WHO-5 = World Health Organization-Five Well-Being Index; FAI = Functional Activities Index; sDQS = short Diet Quality Screener; FQQ = Food Frequency Questionnaire; BDI-II = Beck Depression Inventory-II; MSNQ = Multiple Sclerosis Neuropsychological Questionnaire; MSIS-29 = Multiple Sclerosis Impact Scale-29; MOSS = Medical Outcomes Study Sleep Scale; ESS = Epworth Sleepiness Scale; IRLSS = International Restless Legs Severity Scale; VO_2_max/VO_2_peak = maximal/peak oxygen uptake; mo = months; ↑ = higher value/improvement; ↓ = lower value/worsening

**Table 3:** **Characteristics of secondary studies.**

Author, Year	Type of Review	Aim/Focus	Intervention Category	Included Studies (n)	Key Outcomes	Main Conclusions	Overlap with Primary Studies
Wills & Probst, 2022 [50]	Systematic Review & Meta-analysis	To examine the effectiveness of lifestyle self-management strategies on quality of life and disability in MS	Lifestyle/Self-management	57 studies (*n* = 5830)	MSQOL-54, SF-36, MSIS-29, EDSS, PDDS, relapse rate, MRI	Multicomponent lifestyle self-management improves QOL; dietary-only interventions show no clear benefit; PA effects inconsistent due to heterogeneity	NO
Belveal et al., 2023 [49]	Systematic Review	To evaluate evidence for nontraditional and home-based interventions improving ADLs in MS	Physical/Home-based/Nontraditional	15 studies (*n* = 782)	ADL performance, balance, mobility, fatigue, dexterity	Strong evidence for nontraditional (vestibular, yoga, music) and home-based programs (CBT-online, strengthening, manual dexterity) improving ADLs	NO
Dias et al., 2020 [51]	Systematic Review & Meta-analysis	To determine whether exercise-based telerehabilitation improves pain, physical function and QOL in persons with disabilities	Physical/Telerehabilitation	60 RCTs (48 in meta-analysis; *n* ≈ 4920)	Pain, physical function, QOL (SF-36, other PROMs)	Telerehabilitation is not inferior to face-to-face interventions; effective for pain, physical function and QOL; evidence vs. no-treatment limited	NO
Harrison et al., 2021 [52]	Systematic Review + Network Meta-analysis	To identify which exercise, behavioral, or combined interventions best reduce fatigue in MS	Physical/ Cognitive/Combined	113 studies (*n* = 6909)	Fatigue (MFIS, FSS), follow-up effects	Balance exercises and CBT ranked highest; aerobic/resistive exercise showed moderate benefits; combined interventions promising but under-studied	NO

MS = multiple sclerosis; QOL = quality of life; ADL = activities of daily living; PA = physical activity; CBT = cognitive behavioral therapy; RCT = randomized controlled trial; MFIS = Modified Fatigue Impact Scale; MRI = Magnetic Resonance Imaging; FSS = Fatigue Severity Scale; EDSS = Expanded Disability Status Scale; PDDS = Patient Determined Disease Steps; PROMs = patient-reported outcome measures.

## Data Availability

Not applicable as no new data were generated.

## Appendix A

### Appendix A.1

**Table A1:** **Preferred Reporting Items for Systematic reviews and Meta-Analyses extension for Scoping Reviews (PRISMA-ScR) Checklist****.**

SECTION	ITEM	PRISMA-ScR CHECKLIST ITEM	REPORTED ON PAGE
**TITLE**			
Title	1	Identify the report as a scoping review.	Title page—The title explicitly identifies the manuscript as a scoping review.
**ABSTRACT**			
Structured summary	2	Provide a structured summary that includes (as applicable): background, objectives, eligibility criteria, sources of evidence, charting methods, results, and conclusions that relate to the review questions and objectives.	Abstract—A structured summary is provided including background, objectives, eligibility criteria, sources of evidence, charting methods, results, and conclusions aligned with the review objectives.
**INTRODUCTION**			
Rationale	3	Describe the rationale for the review in the context of what is already known. Explain why the review questions/objectives lend themselves to a scoping review approach.	Introduction—The rationale is described in the context of the existing literature, highlighting fragmentation of evidence and justifying the use of a scoping review approach.
Objectives	4	Provide an explicit statement of the questions and objectives being addressed with reference to their key elements (e.g., population or participants, concepts, and context) or other relevant key elements used to conceptualize the review questions and/or objectives.	Introduction—Explicit objectives are stated using Population, Concept, and Context (PCC) elements.
**METHODS**			
Protocol and registration	5	Indicate whether a review protocol exists; state if and where it can be accessed (e.g., a Web address); and if available, provide registration information, including the registration number.	Materials and Methods—A review protocol was registered on the Open Science Framework (OSF) in August 2025.
Eligibility criteria	6	Specify characteristics of the sources of evidence used as eligibility criteria (e.g., years considered, language, and publication status), and provide a rationale.	Materials and Methods—Eligibility criteria are defined using the PCC framework, including population characteristics, intervention concepts, context, study designs, publication period (2021–2025), and language restrictions.
Information sources	7	Describe all information sources in the search (e.g., databases with dates of coverage and contact with authors to identify additional sources), as well as the date the most recent search was executed.	Materials and Methods—PubMed, Web of Science, and ProQuest databases were searched; the final search was conducted in July 2025.
Search	8	Present the full electronic search strategy for at least 1 database, including any limits used, such that it could be repeated.	Appendix A.2—Full electronic search strategies with Boolean operators and limits are reported.
Selection of sources of evidence	9	State the process for selecting sources of evidence (i.e., screening and eligibility) included in the scoping review.	Materials and Methods—Study selection—Two-stage screening (title/abstract and full text) was conducted independently by two reviewers; disagreements were resolved by consensus or senior reviewer. A PRISMA flow diagram is provided.
Data charting process	10	Describe the methods of charting data from the included sources of evidence (e.g., calibrated forms or forms that have been tested by the team before their use, and whether data charting was done independently or in duplicate) and any processes for obtaining and confirming data from investigators.	Materials and Methods—Data extraction—A structured data charting form was used; data charting was performed independently by two reviewers.
Data items	11	List and define all variables for which data were sought and any assumptions and simplifications made.	Materials and Methods—Data items—All variables extracted from included sources are listed and defined.
Critical appraisal of individual sources of evidence	12	If done, provide a rationale for conducting a critical appraisal of included sources of evidence; describe the methods used and how this information was used in any data synthesis (if appropriate).	Not performed—Critical appraisal was not conducted, consistent with JBI guidance for scoping reviews.
Synthesis of results	13	Describe the methods of handling and summarizing the data that were charted.	Materials and Methods—Synthesis of results—Data were synthesized descriptively and narratively without quantitative pooling.
**RESULTS**			
Selection of sources of evidence	14	Give numbers of sources of evidence screened, assessed for eligibility, and included in the review, with reasons for exclusions at each stage, ideally using a flow diagram.	Results—Overview of the literature search—Numbers of records identified, screened, excluded (with reasons), and included are reported using a PRISMA flow diagram.
Characteristics of sources of evidence	15	For each source of evidence, present characteristics for which data were charted and provide the citations.	Results—Characteristics of included evidence—Study characteristics are presented in Table 1, Table 2 and Table 3 with citations.
Critical appraisal within sources of evidence	16	If done, present data on critical appraisal of included sources of evidence (see item 12).	Not applicable—No critical appraisal was performed.
Results of individual sources of evidence	17	For each included source of evidence, present the relevant data that were charted that relate to the review questions and objectives.	Results—Intervention descriptions and characteristics—Relevant data from each included source are presented.
Synthesis of results	18	Summarize and/or present the charting results as they relate to the review questions and objectives.	Results—Synthesis sections—Findings are summarized in relation to the review objectives.
**DISCUSSION**			
Summary of evidence	19	Summarize the main results (including an overview of concepts, themes, and types of evidence available), link to the review questions and objectives, and consider the relevance to key groups.	Discussion—Main findings are summarized and linked to the review questions and objectives.
Limitations	20	Discuss the limitations of the scoping review process.	Discussion—Limitations—Limitations of the scoping review process are discussed.
Conclusions	21	Provide a general interpretation of the results with respect to the review questions and objectives, as well as potential implications and/or next steps.	Conclusions—General interpretation of results, implications, and future research directions are provided.
**FUNDING**			
Funding	22	Describe sources of funding for the included sources of evidence, as well as sources of funding for the scoping review. Describe the role of the funders of the scoping review.	Funding—This scoping review received no external funding.

### Appendix A.2

**Table A2:** **Boolean structures****.**

Database	Search Strategy	Notes/Platform-Specific Details
PubMed	(“Multiple Sclerosis”[Mesh] OR “Multiple Sclerosis”[tiab]) AND (“Rehabilitation”[Mesh] OR “Rehabilitation”[tiab] OR “Exercise Therapy”[tiab] OR “Physiotherapy”[tiab] OR “Neurorehabilitation”[tiab] OR “Cognitive Behavioral Therapy”[tiab] OR “Cognitive Training”[tiab]) AND (“Quality of Life”[Mesh] OR “Quality of Life”[tiab]) AND (“Lifestyle”[tiab] OR “Health Behavior”[tiab] OR “Self-management”[tiab] OR “Sleep”[tiab] OR “Diet”[tiab] OR “Nutrition”[tiab] OR “Dietary Intervention”[tiab] OR “Nutritional Status”[tiab])	MeSH + free text; filters: 2021–2025, English, full text
Web of Science	TS = (“Multiple Sclerosis”) AND TS = (“Rehabilitation” OR “Exercise Therapy” OR “Physiotherapy” OR “Neurorehabilitation” OR “Cognitive Behavioral Therapy” OR “Cognitive Training”) AND TS = (“Quality of Life”) AND TS = (“Lifestyle” OR “Health Behavior” OR “Self-management” OR “Sleep” OR “Diet” OR “Nutrition” OR “Dietary Intervention” OR “Nutritional Status”)	TS = search in Title, Abstract, Author Keywords; filters: 2021–2025, English
ProQuest	(“Multiple Sclerosis”) AND (“Rehabilitation” OR “Exercise Therapy” OR “Physiotherapy” OR “Neurorehabilitation” OR “Cognitive Behavioral Therapy” OR “Cognitive Training”) AND (“Quality of Life”) AND (“Lifestyle” OR “Health Behavior” OR “Self-management” OR “Sleep” OR “Diet” OR “Nutrition” OR “Dietary Intervention” OR “Nutritional Status”)	Searched in “Anywhere except full text”; filters: 2021–2025, English, peer-reviewed
